# Semantic loss marks early Alzheimer's disease‐related neurodegeneration in older adults without dementia

**DOI:** 10.1002/dad2.12066

**Published:** 2020-08-05

**Authors:** Jet M. J. Vonk, Vincent Bouteloup, Jean‐François Mangin, Bruno Dubois, Frédéric Blanc, Audrey Gabelle, Mathieu Ceccaldi, Cédric Annweiler, Pierre Krolak‐Salmon, Catherine Belin, Thérèse Rivasseau‐Jonveaux, Adrien Julian, François Sellal, Eloi Magnin, Marie Chupin, Marie‐Odile Habert, Geneviève Chêne, Carole Dufouil, and on behalf of the MEMENTO cohort Study Group

**Affiliations:** ^1^ Taub Institute for Research on Alzheimer's Disease and the Aging Brain Department of Neurology College of Physicians and Surgeons Columbia University New York New York USA; ^2^ Julius Center for Health Sciences and Primary Care, Department of Epidemiology University Medical Center Utrecht Utrecht the Netherlands; ^3^ Centre Inserm U1219 d'Epidémiologie et de Développement (ISPED) Bordeaux School of Public Health Institut de Santé Publique Université de Bordeaux Bordeaux France; ^4^ Pole de sante publique Centre Hospitalier Universitaire (CHU) de Bordeaux Bordeaux France; ^5^ CATI Multicenter Neuroimaging Platform Paris France; ^6^ Neurospin CEA Paris Saclay University Gif‐sur‐Yvette France; ^7^ IM2A AP‐HP INSERM UMR‐S975 Groupe Hospitalier Pitié‐Salpêtrière Institut de la Mémoire et de la Maladie d'Alzheimer Institut du Cerveau et de la Moelle épinière Sorbonne Université Paris France; ^8^ Hôpitaux Universitaire de Strasbourg CM2R (Centre Mémoire de Ressource et de Recherche) Hôpital de jour pôle de Gériatrie et CNRS laboratoire ICube UMR 7357 and FMTS (Fédération de Médecine Translationnelle de Strasbourg), team IMIS Strasbourg France; ^9^ Centre Mémoire Ressources Recherche Département de Neurologie CHU Gui de Chauliac Montpellier France; ^10^ Inserm U1061 La Colombière Université de Montpellier Montpellier France; ^11^ CMMR PACA Ouest CHU Timone APHM & Aix Marseille Univ INSERM INS Inst Neurosci Syst Marseille France; ^12^ Department of Geriatric Medicine Angers University Hospital Angers France; ^13^ Angers University Memory Clinic Angers France; ^14^ Research Center on Autonomy and Longevity Angers France; ^15^ UPRES EA 4638 University of Angers Angers France; ^16^ Robarts Research Institute Department of Medical Biophysics Schulich School of Medicine and Dentistry the University of Western Ontario, Ontario London Canada; ^17^ Institut du Vieillissement Centre Mémoire Ressources Recherche de Lyon Hospices civils de Lyon Université Lyon 1, Inserm U1048 Lyon France; ^18^ Service de Neurologie Hôpital Saint‐Louis AP‐HP Paris France; ^19^ Centre Mémoire de Ressources et de Recherche de Lorraine Unité Cognitivo Comportementale CHRU Nancy Laboratoire Lorrain de Psychologie et de Neurosciences de la dynamique des comportements 2LPN EA 7489 Université de Lorraine Nancy France; ^20^ Service de Neurologie CHU La Milétrie Centre Mémoire de Ressources et de Recherche Poitiers France; ^21^ CMRR Département de Neurologie Hôpitaux Civils Colmar France; ^22^ INSERM U‐1118 Université de Strasbourg. Faculté de Médecine Strasbourg France; ^23^ Centre Mémoire Ressources et Recherche (CMRR) service de Neurologie CHRU Besançon Besançon France; ^24^ Neurosciences intégratives et cliniques EA481 Univ. Bourgogne Franche‐Comté Besançon France; ^25^ CNRS INSERM Laboratoire d'Imagerie Biomédicale LIB Sorbonne Université Paris France; ^26^ AP‐HP Hôpital Pitié‐Salpêtrière Médecine Nucléaire Paris France

**Keywords:** Alzheimer's disease, amnestic, biomarkers, category fluency, cognitive aging, cohort studies, letter fluency, MCI, neuroimaging, semantic fluency, verbal fluency

## Abstract

**Objective:**

To assess progression of semantic loss in early stages of cognitive decline using semantic and letter fluency performance, and its relation with Alzheimer's disease (AD)‐specific neurodegeneration using longitudinal multimodal neuroimaging measures.

**Methods:**

Change in verbal fluency was analyzed among 2261 non‐demented individuals with a follow‐up diagnosis of no mild cognitive impairment (MCI), amnestic MCI (aMCI), non‐amnestic MCI (naMCI), or incident dementia, using linear mixed models across 4 years of follow‐up, and relations with magnetic resonance imaging (MRI; n = 1536) and ^18^F‐fluorodeoxyglucose brain positron emission tomography (^18^F‐FDG‐PET) imaging (n = 756) using linear regression models across 2 years of follow‐up.

**Results:**

Semantic fluency declined—fastest in those at higher risk for AD (apolipoprotein E [APOE] e4 carriers, Clinical Dementia Rating score of .5, aMCI, or incident dementia)—while letter fluency did not except for those with incident dementia. Lower baseline semantic fluency was associated with an increase in white matter hyperintensities and total mean cortical thinning over time, and regionally with less hippocampal volume as well as more cortical thinning and reduced ^18^F‐FDG‐PET uptake in the inferior parietal lobule, entorhinal cortex, isthmus cingulate, and precuneus–posterior cingulate area. In contrast, baseline letter fluency was not associated with change in total nor regional neurodegeneration. Whole‐brain neurodegeneration over time was associated with faster decline in both fluencies, while AD‐specific regions were associated with a faster rate of decline in semantic but not letter fluency.

**Interpretation:**

This study provides strong evidence of distinctive degeneration of semantic abilities early on in relation to both cognitive decline and AD‐specific neurodegeneration.

## 1 INTRODUCTION

The preclinical phase of Alzheimer's disease (AD) is not only marked by amyloid and tau accumulation and neurodegeneration,^1^ but also by subtle cognitive changes years before a clinical diagnosis can be established.^2^, ^3^ A diagnostic marker of AD in clinical practice, and supported by observations in research, is a diverging performance pattern of semantic fluency versus letter fluency^4^, ^5^—generating as many words within time limits that start with a specific category or letter, respectively. Cognitively normal individuals typically perform better on semantic fluency than letter fluency,^6^, ^7^ while individuals with manifest clinical AD often show greater impairment in semantic fluency compared to letter fluency.^8^, ^9^, ^10^ The reversal of this pattern over the course of the preclinical to clinical AD process is due to a loss of semantic memory, which is one of the first cognitive domains to become impaired in addition to episodic memory.^11^, ^12^

How the discrepancy between letter and semantic fluency relates to neurobiological change over time in the early stages of AD, however, is relatively unexplored. The cortical signature of neurodegeneration in early stages of AD includes medial‐temporal and temporal‐parietal regions.^13^, ^14^ In the left hemisphere, these regions are particularly associated with semantic processing abilities.^15^, ^16^, ^17^ Correspondingly, semantic fluency has been linked to temporal‐parietal as well as frontal regions, while letter fluency is considered to be relatively confined to inferior frontal regions important for executive functioning.^7^, ^18^ The discrepancy between semantic and letter fluency in clinical AD has been ascribed to semantic deficits that are mediated by neurodegeneration of temporal‐parietal regions.^4^, ^19^ Identifying how the discrepancy in semantic versus letter fluency develops in a preclinical phase, and how it relates to change over time in AD markers of neurodegeneration, will provide better insight into the potential predictive value of this discrepancy in the earliest stages of the AD process. Potential implementations include use of this knowledge to refine the definition of high‐risk individuals for clinical trials aimed at intervention. Additionally, knowledge about the degree of discrepancy and its development over time may be used as a measure of progression in disease‐modifying interventions.

This study aimed to (1) investigate change over time in semantic fluency and letter fluency in the earliest stages of cognitive dysfunction, and (2) examine the relationships of baseline performance and rate of change of semantic versus letter fluency with longitudinal multimodal neuroimaging measures (structural brain magnetic resonance imaging [MRI], and ^18^F‐fluorodeoxyglucose brain positron emission tomography [^18^F‐FDG‐PET] imaging), including localization of associations in temporal‐parietal versus inferior frontal regions and AD‐specific regions. For aim 1, we hypothesized that semantic fluency would decline over time, but letter fluency would stay relatively preserved. For aim 2, we hypothesized that baseline performance and rate of change of semantic fluency, but not letter fluency, would predict follow‐up neuroimaging measures—even when semantic fluency performance is adjusted for letter fluency, which reflects the discrepancy between the two measures.^2^

## 2 METHODS

### 2.1 Participants

Participants were drawn from the Memento cohort, an ongoing multi‐center prospective study on AD and related disorders of 2323 individuals recruited from French memory clinics between 2011 and 2014. Participants were screened at inclusion to be non‐demented (Clinical Dementia Rating [CDR] of ≤.5). All examinations in Memento followed standardized procedures, including neuropsychological assessment, brain MRI, and ^18^F‐FDG‐PET imaging. The cohort, recruitment, design, and procedures are described in detail elsewhere.^20^

**HIGHLIGHTS**

- Semantic fluency declined faster with increased risk for Alzheimer's disease (AD).
- Early‐stage semantic fluency decline correlated with increasing neurodegeneration.
- Semantic fluency was particularly associated with AD‐specific neurodegeneration.
- Letter fluency remained spared and was not associated with neurodegeneration.
- This discrepancy shows distinctive degeneration of semantics early in the AD process.

**RESEARCH IN CONTEXT**

1. **Systematic review**: A systematic literature search was performed in PubMed and Google Scholar. A discrepancy between semantic and letter fluency is often described, but literature on longitudinal patterns of decline in both tasks was sparse, particularly in the earliest stages of the disease process. The link between longitudinal decline in verbal fluency and AD‐specific neurodegeneration remained unknown.
2. **Interpretation**: Our results provide strong evidence of the distinctive degeneration of semantic abilities early on in relation to both cognitive decline over time and AD‐specific neurodegeneration, with relative sparing of lexical retrieval abilities. Our results also confirm theoretical assumptions about the cortical signature of letter versus semantic fluency, and the neurobiological basis of executive and semantic processes underlying these tasks.
3. **Future directions**: The discrepancy in semantic and letter fluency performance may aid preclinical detection of AD. Semantic and letter fluency should be included as basic tasks in every cognitive assessment in aging research.

In the present study, individuals were excluded from analysis if they were missing semantic fluency (n = 15), letter fluency (n = 6), or both (n = 33) at baseline; if they were diagnosed with prevalent dementia at baseline (n = 1); or if they were missing demographic information (n = 7 missing education). A total of 2261 individuals were included in the main analytical sample. Subsamples of individuals who underwent MRI (n = 1542) and ^18^F‐FDG‐PET imaging (n = 740) on two occasions, 2 years apart on average, were also analyzed.

All participants in Memento were fluent French speakers. Measures of sex and level of education were self‐reported; education was categorized as low (completed up until middle school), middle (completed high school), or high (completed a higher‐level diploma). Participants were genotyped for apolipoprotein E (APOE; n = 2146) and categorized as APOE e4 carriers based on the presence of at least one e4 allele.^20^ At baseline, individuals were classified for mild cognitive impairment (MCI) categories following the Petersen criteria^21^ using a full neuropsychological battery (described in detail elsewhere^20^). Participants presented either with MCI (amnestic or non‐amnestic), performing >1.5 standard deviations below age and educational norms in one or more cognitive domains, or with isolated subjective cognitive complaints (SCC) as assessed with a visual analog scale in which participants rated the degree of SCC ranging from not at all to extremely. Similarly, cognitive status at last follow‐up was determined using MCI Petersen criteria and included in addition to no MCI, amnestic MCI (aMCI), non‐amnestic MCI (naMCI) also incident dementia, as assessed by Diagnostic and Statistical Manual of Mental Disorders, 4th edition (DSM‐IV) criteria for dementia and National Institute of Neurological and Communicative Disorders and Stroke/Alzheimer's Disease and Related Disorders Association criteria for AD.^22^, ^23^ The cognitive status at last follow‐up provides future diagnostic information about the trajectory of an individual when assessing cognitive change over time.^24^, ^25^ For example, when cognitive status at last follow‐up is dementia, this information confirms that an individual had pre‐clinical dementia at baseline.

The project was approved by the ethics committee (“Comité de Protection des Personnes Sud‐Ouest et Outre Mer III”) and followed the guidelines of the Declaration of Helsinki. All participants gave written consent.

### 2.2 Cognitive measures

An extensive neuropsychological battery was administered annually, including tasks of memory, language, praxis, visuospatial skills, and executive functioning.^20^ As part of this battery, both letter and semantic fluency were administered. In these verbal fluency tasks, individuals had to generate as many words as possible within 2 minutes that started with the letter p (letter fluency) or within the category animals (semantic fluency). As the Memento Cohort follow‐up is not yet completed, we restricted the data to the first 4 years of follow‐up (time in study <5 years); individuals returned for on average 3.97 visits (SD = 1.47) with an average length of follow‐up of 3.04 years (SD = 1.49). The interval between baseline and follow‐up was on average 1.07 years at visit 2 (SD = .13, n = 1879), 2.08 years at visit 3 (SD = .13, n = 1695), 3.08 years at visit 4 (SD = .15, n = 1490), and 4.08 years at visit 5 (SD = .14, n = 1360).

### 2.3 MRI and ^18^F‐FDG‐PET

Structural MRI was acquired for 1542 individuals and ^18^F‐FDG‐PET imaging for 740 individuals at two consecutive occasions, that is, at baseline and at follow‐up 2 years later. Across the structural MRI metrics, cortical thickness for the whole brain, pars opercularis, inferior parietal lobule, and isthmus cingulate were obtained for all 1542 participants, entorhinal cortex thickness for 1540 participants, parahippocampal thickness for 1537 participants, hippocampal volume 1523 individuals, and white matter hyperintensity (WMH) volume for 1473 individuals. ^18^F‐FDG‐PET standardized uptake value ratio (SUVR) for the pars opercularis was obtained for all 740 participants, and for 739 participants for whole‐brain, inferior parietal lobule, precuneus–posterior cingulate area, and inferior temporal area.

Imaging processes were standardized across memory centers by a specialized neuroimaging team (Centre pour l'Acquisition et le Traitement des Images [CATI]; http://cati-neuroimaging.com/). A description of the processing techniques used to obtain hippocampal volume, WMH, cortical thickness, and 18F‐FDG‐PET imaging SUVR is provided elsewhere.^20^, ^26^ Longitudinal pipelines have been implemented for processing the repeated measures of cortical thickness and ^18^F‐FDG‐PET. ^18^F‐FDG‐PET imaging measured SUVR across the whole brain and for several regions of interest (ROIs); mean uptake for the ROIs was calculated relative to the pons reference region, including partial volume correction. Three structural MRI measures were used for the present study: hippocampal volume, cortical thickness (whole‐brain and of several ROIs), and WMH volume—previous literature strongly suggests that WMH play a role in the pathogenesis of AD.^27^, ^28^

ROI analyses focused on the left hemisphere, given the lateralization of language to this hemisphere.^29^ We analyzed cortical thickness and SUVR in temporal‐parietal and inferior frontal regions previously identified for verbal fluency: the inferior parietal lobule (linked to semantic fluency only) and pars opercularis (linked to both semantic and letter fluency).^7^, ^18^, ^30^ Additionally, we analyzed AD‐specific regions that are affected early in the disease, including volume of the hippocampus, cortical thickness of the entorhinal cortex, parahippocampal gyrus, and isthmus cingulate, and SUVR of the precuneus–posterior cingulate area and inferior temporal area (ie, AD‐specific ROIs inferred from the Alzheimer's Disease Neuroimaging Initiative [ADNI] database by Toussaint et al.^31^).

### 2.4 Statistical analysis

Participant characteristics were extracted using descriptive statistics. Differences in semantic and letter fluency performance were analyzed with general linear models.

To analyze the change of letter and semantic fluency performance over time, linear mixed models were used. Models included either semantic or letter fluency as an outcome, with time in study (in years, starting at 0), age at baseline, an interaction term of time in study and age at baseline (to account for age‐related differential decline^32^, ^33^), sex, education, and practice effect as fixed factors, as well as a random intercept and random slope: $y_{\mathrm{ij}}=\left(\beta_{0}{+}_{0i}\right)+\left(\beta_{1}{+}_{1i}\right)\cdot \mathrm{tim}e_{\mathrm{ij}}+\;\beta_{2}\cdot \mathrm{ag}e_{i}+\;\beta_{3}\cdot \mathrm{tim}e_{\mathrm{ij}}\cdot \mathrm{ag}e_{i}+\beta_{4}\cdot \mathrm{se}x_{i}\;+\;\beta_{5}\cdot \mathrm{educ}\mathrm{atio}n_{i}\;+\;\beta_{6}\cdot \mathrm{prac}\mathrm{tic}e_{i}\;+\;\varepsilon_{\mathrm{ij}}$. Models with an interaction term of time in study and age at baseline fitted better than those without this interaction for both semantic (Akaike information criterion [AIC] with 56609.01 vs without 56615.98) and letter fluency (AIC with 52805.31 vs without 52811.59). Semantic and letter fluency were standardized by subtracting the test's mean score of the study sample at baseline from each individual's score, and dividing by the study sample's standard deviation at baseline. To report effects of fluency performance at baseline (ie, intercept) and across time (eg, slope), age was centered and sex and education were treated as covariates as opposed to factors to reflect performance of the average participant in all models. Practice effects, often representing reduced anxiety on successive testing occasions, were modeled using an indicator variable being the square root of the number of prior testing occasions.^34^ This variable represents that practice effects are present throughout follow‐up, but that the largest effects occur after the first exposure and gradually diminish across follow‐up visits. The intraclass correlation coefficient (ICC; variance of a random effect/total random variance) was calculated for intercept and slope in the overall models of semantic and letter fluency to indicate how much variance was explained by the random effects. We have also reported the correlation coefficient between intercept and slope (I‐S corr) for the overall models of semantic and letter fluency, which reflects the degree to which individuals with a higher baseline decline at a faster rate than those with a lower baseline.

In separate models, analyses were stratified by cognitive status at follow‐up (no MCI, aMCI, naMCI, incident dementia), education (low, middle, high), sex (men/women), CDR score at inclusion (0/.5), and APOE status (e4+/e4–). Differences in slopes across groups were formally tested with an interaction of each variable with time in study; we did not include a triple interaction of years in study, group, and age in these models because the interaction was non‐significant and worsened model fit for all models. Interactions were performed with the following reference groups: no MCI and naMCI for cognitive status, low and middle for education, men for sex, 0 for CDR score, and e4– for APOE status.

To analyze the relationship of baseline performance and rate of change (ie, slope) of semantic versus letter fluency with longitudinal multimodal neuroimaging measures, multiple linear regression models were used. Individual rates of change were extracted from the previously estimated linear mixed models (ie, individual estimates for time in study regressed on fluency performance), adjusted for age, age x time in study, sex, education, and practice effect. The multiple linear regression models analyzed whether semantic or letter fluency (baseline and rate of change) were predictors of follow‐up neuroimaging, which was autoregressed on neuroimaging at baseline. Models of baseline performance adjusted for age, sex, education, and the interaction of magnet strength (1.5T or 3T) with the manufacturer (Siemens, Phillips, or GE Healthcare) to account for center‐specific differences. Models of rate of change adjusted for the interaction of magnet strength with the manufacturer, as the slopes were previously adjusted for age, sex, education, and practice effect. Differences across groups based on cognitive status at follow‐up were formally tested with an interaction of cognitive status with fluency performance.

Subsequent models added the other fluency measure as a covariate to analyze the discrepancy between letter and semantic fluency.^2^ Autoregression accounts for baseline levels but may sometimes induce bias;^35^ therefore, the same analyses were run in models using difference scores between baseline and follow‐up neuroimaging measurements (however, this method does not account for the initial level of the dependent variable). Both options to model change yielded similar patterns, and below we report the results of autoregression.

The multiple linear regression models were performed for whole‐brain measures of neurodegeneration (ie, WMH, total mean cortical thickness, and total ^18^F‐FDG‐PET SUVR) and for regional measures to investigate localization of effects (ie, hippocampal volume, cortical thickness of the entorhinal cortex, parahippocampal gyrus, and isthmus cingulate, and SUVR of the precuneus–posterior cingulate area and inferior temporal area).

Analyses were performed in R version 3.6.0 using the car, nlme, dplyr, tableone, ggplot2, and directlabels packages.

## 3 RESULTS

### 3.1 Participant characteristics

Demographic information, cognitive status, CDR score, and APOE e4 status for the overall sample (N = 2261), as well as for the subsets who underwent MRI (n = 1542) and ^18^F‐FDG‐PET imaging (n = 740) on two occasions, are presented in Table 1. In the overall sample, mean semantic fluency performance at baseline—adjusted for age, sex, and education—was higher than mean letter fluency performance (F[1, 2256] = 150.187, *P* < .001), and this difference in performance remained at visit 2 (F[1, 1871] = 152.635, *P* < .001), visit 3 (F[1, 1690] = 135.521, *P* < .001), visit 4 (F[1, 1486] = 83.047, *P* < .001), and visit 5 (F[1, 1356] = 76.511, *P* < .001).

**TABLE 1:** Participants’ characteristics

		Overall sample N = 2261	Subsample MRI n = 1523	Subsample ^18^F‐FDG‐PET n = 740
Age (mean, SD)		70.8 (8.7)	70.5 (8.3)	71.1 (8.1)
Sex (n, % women)		1403 (62.1)	920 (60.4)	423 (57.2)
Education (n, %)	Low	282 (12.5)	162 (10.6)	84 (11.4)
	Middle	1049 (46.4)	698 (45.8)	322 (43.5)
	High	930 (41.1)	663 (43.5)	334 (45.1)
CDR (n, % score .5)		1343 (59.7)	852 (56.2)	375 (50.7)
APOE e4 (n, % carrier)		640 (29.8)	445 (30.5)	219 (30.6)
Cognitive status at baseline (n, %)	no MCI	352 (15.6)	263 (17.3)	136 (18.4)
	aMCI	1182 (52.3)	760 (49.9)	332 (44.9)
	naMCI	725 (32.1)	500 (32.8)	272 (36.8)
Cognitive status at last follow‐up (n, %)	no MCI	586 (25.9)	464 (30.5)	231 (31.2)
	aMCI	728 (32.2)	402 (26.4)	171 (23.1)
	naMCI	729 (32.3)	505 (33.2)	274 (37.0)
	incident dementia	216 (9.6)	152 (10.0)	64 (8.6)
Average follow‐up time by cognitive status at last follow‐up (n, %)	no MCI	3.63 (1.05)	3.90 (.52)	3.97 (.36)
	aMCI	2.79 (1.61)	3.68 (.78)	3.74 (.73)
	naMCI	3.33 (1.33)	3.85 (.57)	3.89 (.52)
	incident dementia	1.33 (1.09)	1.52 (1.04)	1.42 (1.02)
Semantic fluency (mean, SD)		28.29 (8.73)	28.97 (8.63)	29.66 (8.52)
Letter fluency (mean, SD)		20.37 (7.18)	20.81 (7.21)	21.18 (7.12)
Birth country (n, %)	France	1957 (86.6)	1342 (88.1)	657 (88.8)
	Algeria	96 (4.2)	61 (4.0)	24 (3.2)
	Morocco	39 (1.7)	29 (1.9)	22 (3.0)
	Tunisia	25 (1.1)	15 (1.0)	10 (1.4)
	Other	144 (6.4)	76 (5.0)	27 (3.6)
Native language French (n, %)		114 (5.0)	69 (4.5)	23 (3.1)

Abbreviations: ^18^F‐FDG‐PET, ^18^F‐fluorodeoxyglucose brain positron emission tomography; aMCI, amnestic MCI; APOE, apolipoprotein E; CDR, Clinical Dementia Rating; MCI, mild cognitive impairment; MRI, magnetic resonance imaging; naMCI, non‐amnestic MCI; SD, standard deviation.

### 3.2 Longitudinal cognitive change

Semantic fluency declined across annual assessments (B = −.044, SE = .015, *P* = .003; ICC intercept = .674, ICC slope = .012, I‐S corr = −.025), while letter fluency did not (B = .002, SE = .014, *P* = .871; ICC intercept = .689, ICC slope = .010, I‐S corr = −.025). Decline over time became stronger with older age in both semantic fluency (B = −.002, SE = .001, *P* < .001) and letter fluency (B = −.002, SE = .001, *P* < .001).

**FIGURE 1:**
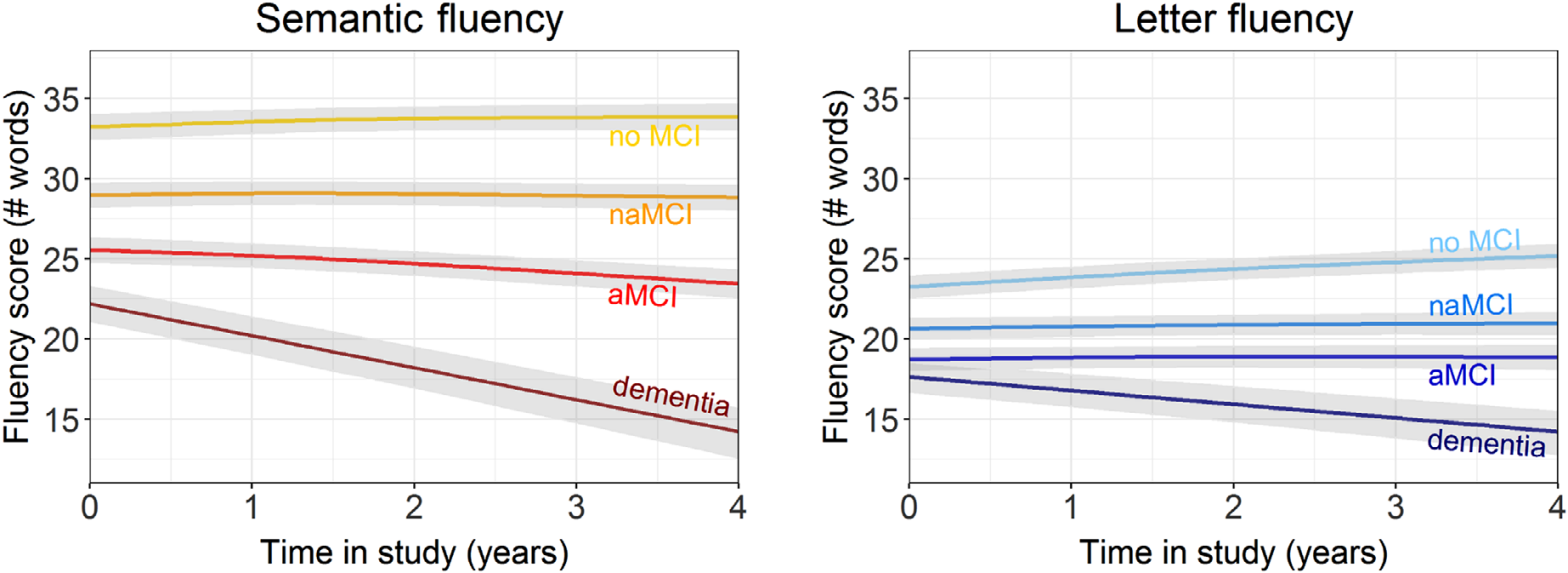
Trajectories of semantic and letter fluency across diagnosis at follow‐up, including 95% confidence interval bands

Table 2 displays estimates in stratified analyses by cognitive status at last follow‐up, education, sex, CDR score at inclusion, and APOE status. Testing differences between groups showed that in both fluencies baseline performance (semantic: χ^2^ = 641.854, *P* < .001; letter: χ^2^ = 235.708, *P* < .001) and change over time (semantic: χ^2^ = 109.282, *P* < .001; letter: χ^2^ = 64.171, *P* < .001) differed across cognitive status at last follow‐up. Specifically, in models of semantic fluency, baseline performance was lower in aMCI (B = −.748, SE = .046, *P* < .001), naMCI (B = −.410, SE = .046, *P* < .001), and dementia (B = −1.075, SE = .069, *P* < .001) compared to no MCI, and lower in aMCI (B = −.339, SE = .043, *P* < .001) and dementia (B = −.665, SE = .066, *P* < .001) compared to naMCI. Rate of decline in semantic fluency was faster in aMCI (B = −.082, SE = .012, *P* < .001) and dementia (B = −.263, SE = .031, *P* < .001) but not naMCI (B = −.020, SE = .012, *P* = .088) compared to no MCI, and faster in aMCI (B = −.062, SE = .012, *P* < .001) and dementia (B = −.243, SE = .030, *P* < .001) compared to naMCI. In models of letter fluency, baseline performance was lower in aMCI (B = −.500, SE = .049, *P* < .001), naMCI (B = −.322, SE = .048, *P* < .001), and dementia (B = −.640, SE = .072, *P* < .001) compared to no MCI, and lower in aMCI (B = −.178, SE = .046, *P* < .001) and dementia (B = −.318, SE = .069, *P* < .001) compared to naMCI. Rate of decline in letter fluency was faster in aMCI (B = −.064, SE = .012, *P* < .001), naMCI (B = −.054, SE = .011, *P* < .001), and dementia (B = −.201, SE = .030, *P* < .001) compared to no MCI, and was faster in dementia (B = −.147, SE = .030, *P* < .001) compared to naMCI, but did not differ between aMCI (B = −.010, SE = .011, *P* = .395) and naMCI. Figure 1 shows trajectories of both fluencies across cognitive status at last follow‐up.

**TABLE 2:** Intercept and slope effects across diagnosis at last follow‐up, strata of education, sex, CDR score, and APOE e4 status

		Semantic fluency		Letter fluency	
		Intercept (SE)	Slope (SE)^1^	Intercept (SE)	Slope (SE)^1^
Diagnosis	No MCI	−.126 (.130)	−.027 (.028)	−.668 (.135)	−.008 (.027)
	aMCI	−.735 (.116)	−.113 (.026)***	−1.043 (.126)	.015 (.026)
	naMCI	−.383 (.122)	−.034 (.025)	−.765 (.132)	−.027 (.024)
	Dementia	−1.047 (.220)	−.288 (.085)***	−.844 (.236)	−.101 (.100)
Education	Low	−.285 (.175)	.040 (.039)	−0.673 (.195)	.025 (.038)
	Middle	−.175 (.092)	−.068 (.021)**	−.452 (.094)	.018 (.021)
	High	−.057 (.096)	−.039 (.024)	−.023 (.093)	−.018 (.023)
Sex	Men	−.423 (.069)	−.038 (.024)	−.672 (.069)	.007 (.023)
	Women	−.612 (.047)	−.046 (.019)*	−.617 (.048)	< −.001 (.018)
CDR score	0	−.371 (.094)	−.017 (.023)	−.710 (.098)	−.029 (.022)
	.5	−.688 (.086)	−.064 (.023)**	−.911 (.086)	.026 (.021)
APOE status	e4−	−.645 (.090)	−.029 (.018)	−.946 (.090)	.008 (.017)
	e4+	−.789 (.139)	−.084 (.028)**	−.917 (.140)	−.017 (.028)

*Note*. ^1^Significance of slope effects: ^*^ *P* < .05, ^**^ *P* < .01, ^***^ *P* < .001. Models are adjusted for age at baseline, age at baseline x time in study, sex, and education—unless sex or education was used to stratify—and models of letter fluency were additionally adjusted for time in study x time in study

Abbreviations: aMCI, amnestic MCI; APOE, apolipoprotein E; CDR, Clinical Dementia Rating; ICC, intraclass correlation coefficient; I‐S Corr, intercept‐slope correlation; MCI, mild cognitive impairment; naMCI, non‐amnestic MCI; SE, standard error.

**FIGURE 2:**
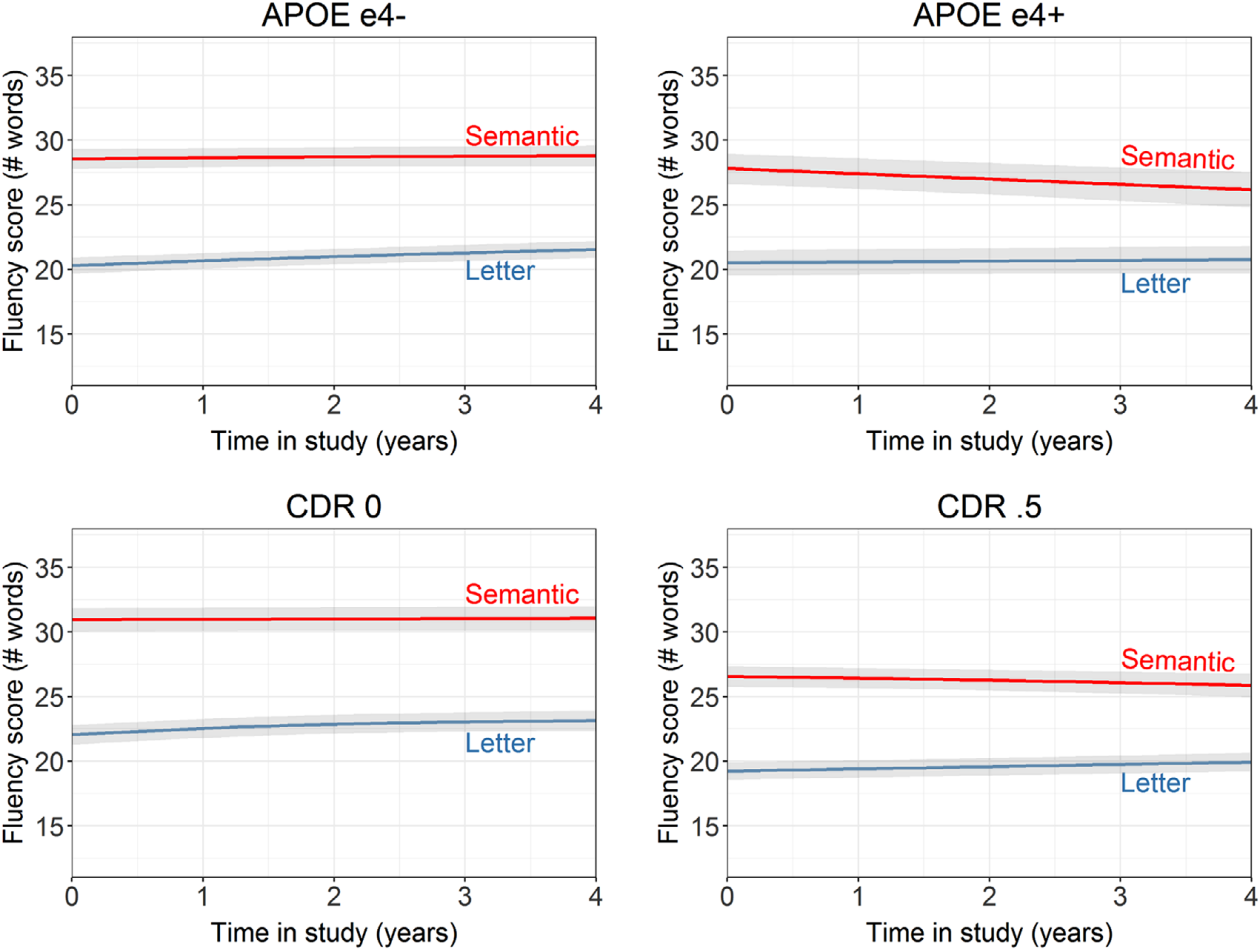
Trajectories of semantic and letter fluency across strata of apolipoprotein E e4 status and baseline Clinical Dementia Rating score, including 95% confidence interval bands

Baseline performance of semantic fluency was lower (B = −.092, SE = .042, *P* = .029) and the rate of decline faster (B = −.049, SE = .011, *P* < .001) in APOE e4 carriers compared to non‐carriers. Similarly, baseline performance of semantic fluency was lower (B = −.429, SE = .037, *P* < .001) and the rate of decline faster (B = −.033, SE = .010, *P* < .001) in individuals with a CDR of .5 compared to those with a CDR of 0 at baseline. Baseline performance of letter fluency did not differ across APOE e4 status (B = .035, SE = .042, *P* = .399), but APOE e4 carriers had a faster rate of decline compared to non‐carriers (B = −.037, SE = .011, *P* < .001). Performance of letter fluency was lower at baseline (B = −.309, SE = .036, *P* < .001) and had a faster rate of decline (B = −.019, SE = .009, *P* = .045) in individuals with a CDR of .5 compared to those with a CDR of 0 at baseline. Figure 2 shows the trajectories of semantic and letter fluency across strata of APOE e4 status and baseline CDR score.

Baseline performance of semantic fluency differed across education groups (χ^2^ = 253.040, *P* < .001), but change over time did not (χ^2^ = 1.744, *P* = .418). In detail, middle (B = .378, SE = .060, *P* < .001) and high (B = .839, SE = .061, *P* < .001) education groups had higher baseline performance than the low education group, and the high education group had higher baseline performance than the middle education group (B = .461, SE = .040, *P* < .001). Baseline performance of semantic fluency did not differ across sex (B = .073, SE = .039, *P* = .059), while rate of decline was faster in men than women (B = .022, SE = .010, *P* = .025). Similarly, baseline performance of letter fluency differed across education groups (χ^2^ = 350.932, *P* < .001), but change over time did not (χ^2^ = .891,*P* = .641). In detail, middle (B = .551, SE = .060, *P* < .001) and high (B = 1.029, SE = .061, *P* < .001) education groups had higher baseline performance than the low education group, and the high education group had higher baseline performance than the middle education group (B = .478, SE = .040, *P* < .001). Baseline performance of letter fluency was higher in women than men (B = .179, SE = .039, *P* < .001), but change over time did not differ across sex (B = .013, SE = .010, *P* = .166).

### 3.3 Prediction of follow‐up neuroimaging by baseline fluency performance

**FIGURE 3:**
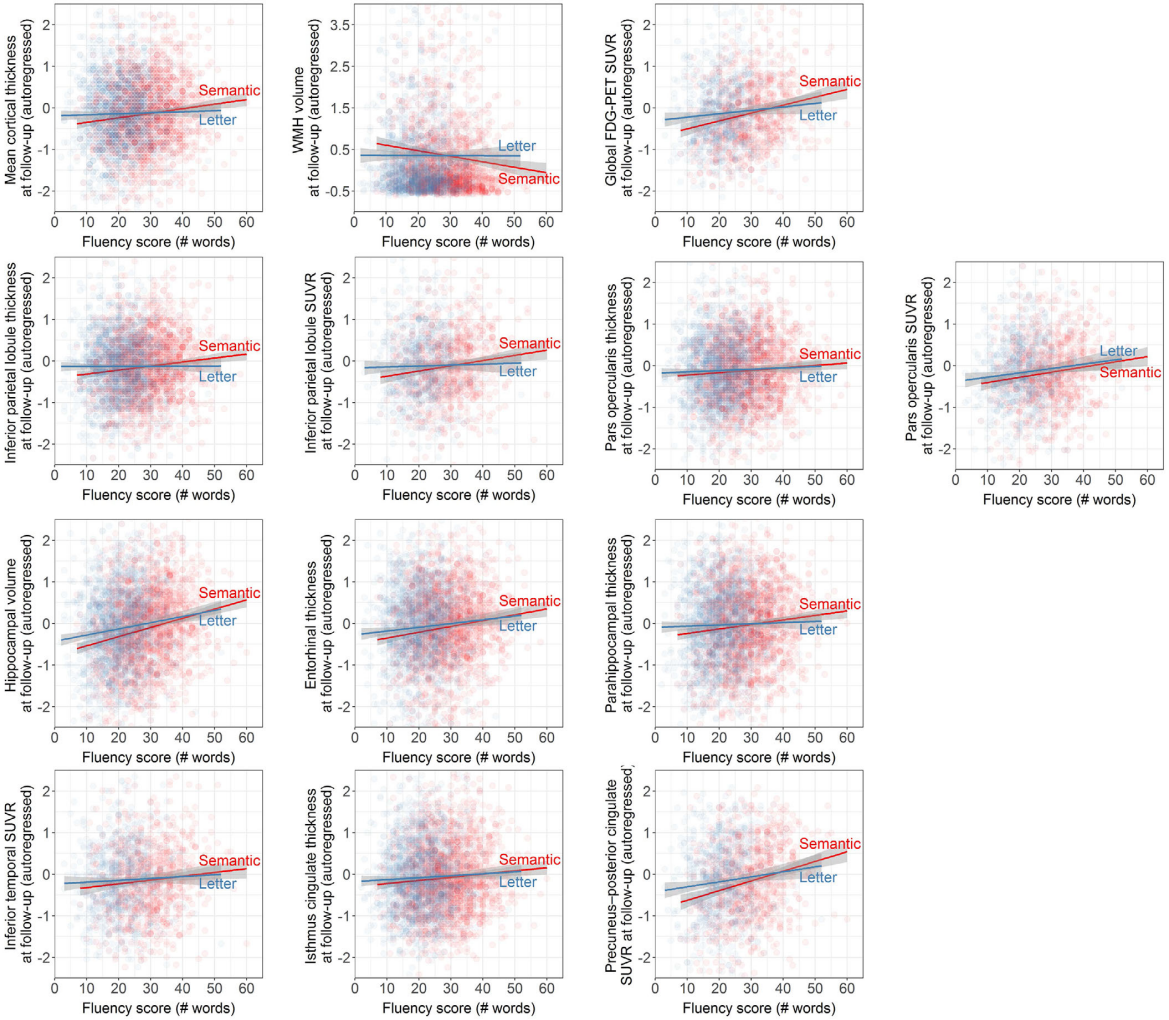
Relationships of baseline performance in semantic and letter fluency with neurodegeneration

Lower baseline semantic fluency, but not letter fluency, was associated with more neurodegeneration over time in whole‐brain neuroimaging measures of total mean cortical thickness and WMH volume, but not total SUVR on ^18^F‐FDG‐PET (Table 3 and Figure 3). These patterns remained similar when modeling the discrepancy between the two fluency measures by adjusting for each other as covariates. Regional measures showed a similar pattern of semantic fluency but not letter fluency predicting neurodegeneration over time in both unadjusted and adjusted models for inferior parietal thickness and SUVR, hippocampal volume, entorhinal thickness, isthmus cingulate thickness, and precuneus–posterior cingulate SUVR. In the unadjusted model with pars opercularis thickness, letter fluency was not associated with change in cortical thickness, but semantic fluency was. However, in a model that adjusted for the other fluency measure, the effect of letter fluency for this ROI was strongly reduced and the association between semantic fluency was slightly reduced. There was no relationship between change in ^18^F‐FDG‐PET SUVR in the pars opercularis or inferior temporal area, nor parahippocampal thickness, with letter or semantic fluency in unadjusted and adjusted models. Testing the associations of semantic and letter fluency with change in neurodegeneration across cognitive status at last follow‐up showed that the strength of the relationships was stronger for those who developed incident dementia compared to those without MCI at follow‐up for whole‐brain measures of total mean cortical thickness and WMH, but did not differ across cognitive status groups in ROI analyses (Table S1 in supporting information).

**TABLE 3:** Relationships of verbal fluency performance (baseline and trajectory) with global and regional neurodegeneration over time in the overall sample

		*Baseline fluency*		*Change in fluency*	
	Fluency	Unadjusted	Adjusted	Unadjusted	Adjusted
Global neurodegeneration					
Total cortical thickness	Semantic	**.005 (.002), *P* = .003**	**.006 (.002), *P* = .004**	**.119 (.032), *P* < .001**	**.094 (.034), *P* = .005**
	Letter	.002 (.002), *P* = .432	−.002 (.002), *P* = .478	**.154 (.045), *P* = .001**	**.111 (.048), *P* = .020**
WMH volume	Semantic	−**.003 (.001), *P* = .038**	−**.003 (.001), *P* = .035**	−**.059 (.023), *P* = .011**	−.040 (.024), *P* = .098
	Letter	−.001 (.001), *P* = .638	.001 (.002), *P* = .548	−**.099 (.032), *P* = .002**	−**.081 (.034), *P* = .018**
^18^F‐FDG‐PET SUVR	Semantic	−.002 (.003), *P* = .536	< .001 (.004), *P* = .973	.100 (.055), *P* = .071	.087 (.058), *P* = .135
	Letter	−.005 (.004), *P* = .159	−.006 (.004), *P* = .206	.103 (.083), *P* = .212	.063 (.087), *P* = .468
Regional neurodegeneration					
*Inferior parietal lobule*:	Semantic	**.006 (.002), *P* = .006**	**.006 (.002), *P* = .006**	**.155 (.037), *P* < .001**	**.131 (.039), *P* = .001**
Cortical thickness	Letter	.002 (.002), *P* = .479	−.002 (.003), *P* = .478	**.168 (.052), *P* = .001**	.107 (.055), *P* = .052
*Inferior parietal lobule*:	Semantic	**.007 (.003), *P* = .030**	**.009 (.004), *P* = .020**	**.118 (.057), *P* = .038**	.101 (.060), *P* = .091
^18^F‐FDG‐PET SUVR^a^	Letter	.001 (.004), *P* = .795	−.004 (.004), *P* = .398	.128 (.085), *P* = .134	.080 (.089), *P* = .370
*Pars opercularis*:	Semantic	**.005 (.002), *P* = .018**	.004 (.002), *P* = .088	.016 (.037), *P* = .673	−.010 (.039), *P* = .808
Cortical thickness	Letter	.004 (.002), *P* = .069	.002 (.003), *P* = .438	**.106 (.053), *P* = .047**	**.110 (.056), *P* = .050**
*Pars opercularis*:	Semantic	.005 (.003), *P* = .114	.004 (.003), *P* = .207	**.106 (.051), *P* = .037**	**.113 (.053), *P* = .034**
^18^F‐FDG‐PET SUVR	Letter	.004 (.004), *P* = .315	.001 (.004), *P* = .752	.017 (.076), *P* = .822	−.036 (.08), *P* = .652
*Hippocampal volume*	Semantic	**.003 (.001), *P* = .011**	**.003 (.001), *P* = .011**	**.073 (.019), *P* < .001**	**.060 (.020), *P* = .003**
	Letter	.001 (.001), *P* = .531	−.001 (.001), *P* = .514	**.085 (.027), *P* = .002**	**.058 (.029), *P* = .044**
*Entorhinal cortex*:	Semantic	**.006 (.001), *P* < .001**	**.006 (.002), *P* < .001**	**.088 (.026), *P* = .001**	**.082 (.027), *P* = .002**
Cortical thickness	Letter	.003 (.002), *P* = .059	< .001 (.002), *P* = .865	.060 (.037), *P* = .103	.022 (.038), *P* = .558
*Parahippocampal gyrus*:	Semantic	.001 (.001), *P* = .242	.001 (.001), *P* = .302	**.090 (.020), *P* < .001**	**.082 (.021), *P* < .001**
Cortical thickness	Letter	.001 (.001), *P* = .581	< .001 (.002), *P* = .990	**.074 (.028), *P* = .009**	.037 (.030), *P* = .222
*Inferior temporal area* a:	Semantic	.003 (.003), *P* = .360	.004 (.004), *P* = .246	.091 (.056), *P* = .105	.081 (.059), *P* = .171
^18^F‐FDG‐PET SUVR	Letter	−.001 (.004), *P* = .807	−.003 (.004), *P* = .452	.086 (.084), *P* = .307	.048 (.088), *P* = .587
*Isthmus cingulate*:	Semantic	**.003 (.001), *P* = .024**	**.004 (.002), *P* = .011**	**.057 (.025), *P* = .023**	.046 (.027), *P* = .086
Cortical thickness	Letter	<.001 (.002), *P* = .982	−.002 (.002), *P* = .227	**.072 (.036), *P* = .045**	.051 (.038), *P* = .182
*Precuneus–posterior cingulate area* a: ^18^F‐FDG‐PET SUVR	Semantic	**.007 (.003), *P* = .012**	**.009 (.003), *P* = .005**	**.108 (.049), *P* = .028**	.092 (.052), *P* = .076
	Letter	<.001 (.003), *P* = .986	−.005 (.004), *P* = .203	.122 (.074), *P* = .098	.079 (.077), *P* = .305

*Note*. Cells represent beta estimate (standard error), *P*‐value—values in bold represent that a lower baseline fluency performance or faster rate of fluency decline related to more neurodegeneration.

a Disease‐specific 18F‐FDG‐PET SUVR ROIs inferred from the ADNI database^31^; ROIs are in the left hemisphere; models are adjusted for age, sex, and education.

Abbreviations: ^18^F‐FDG‐PET, ^18^F‐fluorodeoxyglucose brain positron emission tomography; SUVR, standardized uptake value ratio; WMH, white matter hyperintensities.

### 3.4 Prediction of follow‐up neuroimaging by change in fluency performance

Table 3 presents the relationships of follow‐up whole‐brain neurodegeneration and ROIs with change in fluency performance (Figure S1 in supporting information). Faster rate of decline in both semantic and letter fluency was related to more total mean cortical thinning over time in unadjusted and adjusted models. This relationship for semantic fluency was stronger in aMCI and incident dementia groups compared to the naMCI group, while the relationship with letter fluency was stronger in the naMCI group compared to the no MCI group (Table S1). Faster rate of decline in both semantic and letter fluency was also related to an increase in WMH volume over time in unadjusted models and attenuated for semantic fluency in adjusted models; the strength of the relationship did not differ across groups of cognitive status at last follow‐up. Change in whole‐brain ^18^F‐FDG‐PET SUVR was not associated with a faster rate of decline in semantic or letter fluency in unadjusted or adjusted models.

Regionally, faster rate of decline in both semantic and letter fluency was associated with more cortical thinning over time in the inferior parietal lobule in unadjusted models, and this relationship attenuated for letter fluency but not semantic fluency in adjusted models. The relationship of semantic fluency was stronger for the aMCI and dementia groups compared to the naMCI group, and the relationship for letter fluency was stronger for the naMCI group compared to the no MCI group. Less inferior parietal lobule SUVR over time was associated with faster rate of decline in semantic but not letter fluency in unadjusted models, and this relationship attenuated in the adjusted model. Rate of decline in letter but not semantic fluency was associated with more cortical thinning over time in the pars opercularis in adjusted and unadjusted models, but we observed the opposite pattern for SUVR in the pars opercularis. Rate of decline of both semantic and letter fluency was associated with a decline in hippocampal volume over time, but only for semantic fluency was this relationship stronger for the aMCI group compared to the naMCI group. A faster rate of decline in semantic fluency but not letter fluency was associated with more cortical thinning over time in the entorhinal cortex in adjusted and unadjusted models. This relationship was stronger for the aMCI group than the no MCI group. A faster rate of decline in both semantic and letter fluency was related to more cortical thinning over time in the parahippocampal gyrus in unadjusted models, but this relationship only remained present for semantic fluency in adjusted models. Change in SUVR in the inferior temporal area did not show a relationship with rate of decline in semantic or letter fluency. Faster rate of decline in both semantic and letter fluency was related to more cortical thinning over time in the isthmus cingulate in unadjusted models, and this relationship attenuated only for letter fluency in adjusted models; the relationship for semantic fluency was stronger for the aMCI than naMCI group. Last, less SUVR in the precuneus–posterior cingulate area over time was related to a faster rate of decline in semantic fluency but not letter fluency in unadjusted models, and this relationship attenuated in adjusted models.

## 4 DISCUSSION

We demonstrated the progression of semantic impairment at baseline and semantic decline over time in early stages of cognitive decline and its relation with AD biomarkers of neurodegeneration, including total mean cortical thickness, WMH, hippocampal volume, inferior parietal lobule cortical thickness and^18^F‐FDG‐PET SUVR, entorhinal cortical thickness, isthmus cingulate cortical thickness, and precuneus–posterior cingulate area ^18^F‐FDG‐PET SUVR. A large number of both whole‐brain and regional neurodegeneration measures showed that the relationship between the rate of decline in semantic fluency (but not letter fluency) and more AD‐related neurodegeneration over time was stronger in the aMCI than naMCI group—aMCI is known to have a high likelihood of progressing to AD whereas naMCI has a higher likelihood of progressing to non‐AD dementia.^36^ The findings confirm theoretical assumptions about the cortical signature of letter versus semantic fluency, and the neurobiological basis of executive and semantic processes underlying these tasks. This study provides strong evidence of the distinctive degeneration of semantic abilities early on in relation to both AD‐specific cognitive decline over time and AD‐specific neurodegeneration, with relative sparing of lexical retrieval and executive function abilities.

Individuals with manifest clinical AD dementia are often observed to have better letter than semantic fluency, the opposite pattern of cognitively normal adults.^8^, ^9^, ^10^ In our data, individuals with subjective complaints or naMCI exhibited a more similar pattern to cognitively normal adults, namely better semantic than letter fluency at baseline—in line with the findings by Rinehardt et al.^37^—with relatively little change over time. Individuals with aMCI, however, showed a pattern of stronger decline in semantic fluency with relatively stable letter fluency; individuals with incident dementia showed decline over time in both fluencies, yet faster decline for semantic than letter fluency. This longitudinal observation in those at highest risk for AD exemplifies the progressive process of loss of semantic knowledge in the earliest stages of AD,^11^, ^12^ while other cognitive domains, like executive functions of lexical retrieval, are less affected.

The robustness of this pattern is shown in every stratum we analyzed (Table 2). That the pattern is stronger in those who are at higher risk for AD dementia based on higher CDR score, APOE e4 positivity, or a cognitive status of aMCI or incident dementia at last follow‐up (as opposed to subjective complaints only) corresponds with a prevalence study on the discrepancy of the verbal fluency pattern in AD by Sherman and Massman.^38^ While two thirds of their 217 individuals with AD showed the expected reversal of the pattern, the 33% who did not show this pattern at the moment of examination scored significantly higher on the Mini‐Mental State Examination, suggesting a less severe stage of AD—potentially these individuals had not reached the state yet in which the pattern would be reversed. Performance on semantic fluency also decreased more strongly in men than women, in line with previously reported sex differences in semantic fluency decline.^39^

Semantic fluency decline in individuals at high risk for AD in combination with relatively maintained levels of letter fluency over time results in intersection of these trajectories at some point. As the discrepancy between semantic and letter fluency was absent in individuals with a follow‐up cognitive status of subjective decline or naMCI, this differential decline in semantic versus letter fluency marks the earliest stages of cognitive decline in AD. Therefore, verbal fluency tasks can be used to identify who is at risk for AD. While the fluency trajectories over time seem to differentiate groups based on their likelihood of future AD, an isolated observation of semantic versus letter fluency performance at one time point may not be informative enough to identify semantic impairment in the earliest stages of AD; more sensitive measurements of semantic fluency are needed. A promising avenue to exploit semantic fluency performance is to analyze item‐level data. Classically, analyses of cluster and switches have already been shown to add above and beyond total number of items in detecting MCI^40^, ^41^ and very mild AD,^42^ and usage of psycholinguistic variables has demonstrated its value in distinguishing cognitively normal individuals at increased genetic risk of AD dementia by virtue of carrying an APOE e4 allele.^43^ More research in this area should be encouraged, as the present study adds more evidence to the growing body of research on semantic breakdown in the very early stages of AD. This knowledge may be used as a sensitive clinical marker in preclinical AD.^44^

The emerging discrepancy in semantic versus letter fluency in the early stages of AD was related to the neurodegenerative processes of the disease, including hippocampal atrophy, whole‐brain and regional cortical thinning, regional ^18^F‐FDG‐PET SUVR, and progression of small vessel disease as measured by WMH.^45^ The discrepancy in semantic versus letter fluency may have future potential as an inexpensive and easy‐to‐administer proxy for AD‐related neurodegenerative processes otherwise detected on expensive and time‐consuming brain scans, but this application for clinical use should be further investigated.

Semantic fluency is considered to call on two cognitive abilities, namely semantic processing and executive functioning, and letter fluency is considered to call predominantly on executive functioning.^46^ By controlling for letter fluency when analyzing semantic fluency, and vice versa, the effects of the semantic processing versus executive functioning components can be isolated to a certain extent,^2^ to show that these two fluency tasks tap distinctive cognitive processes.^41^ Our results display the hybrid character of both tasks in their cortical signature: The ROI analyses confirm previous findings of letter fluency mediating inferior frontal regions and semantic fluency mediating both inferior frontal and temporal‐parietal regions.^7^, ^18^ The impairment in semantic fluency in clinical AD is thought to be mediated by neurodegeneration of temporal‐parietal regions.^4^, ^19^ Our study showed that indeed, more cortical thinning and lower cerebral metabolic rates of glucose over time in the inferior parietal lobule, a region important for semantic processing,^15^, ^16^, ^17^ are related to a lower baseline score on semantic fluency and a faster rate of decline in semantic fluency.^47^ Importantly, baseline letter fluency and the adjusted rate of change were not related to change in these measures in the inferior parietal lobule. The pars opercularis in the inferior frontal gyrus was related to semantic fluency at baseline (cortical thickness) and semantic fluency's rate of decline (^18^F‐FDG‐PET SUVR), and the rate of decline in letter fluency (cortical thickness). The strength of association of both baseline semantic and letter fluency with the pars opercularis attenuated after adjustment for the other measure, which may suggest that the relationship of each fluency task with this ROI may be driven by the shared executive component of these tasks. These results build on top of prior experiments with transcranial magnetic stimulation to pull apart semantic representation versus controlled retrieval in semantic tasks and identify the functional specialization of the cortex with regard to semantic abilities.^17^ However, the relationships of the rate of change of semantic fluency (cortical thickness) and letter fluency (^18^F‐FDG‐PET SUVR) with this region did not attenuate after adjustment. The differential results across baseline performance and rate of decline, as well as across cortical thickness and ^18^F‐FDG‐PET SUVR, render any interpretation precarious, and further research is needed to decipher the cross‐sectional and longitudinal relationships of both fluency metrics with the inferior frontal gyrus.

By investigating longitudinal change in neuroimaging measures in relation to both baseline and longitudinal change in cognition, this study goes beyond previous correlational studies of the brain‐behavior relationship.^48^ This knowledge can contribute to bringing forward the ability to identify individuals at high risk of developing clinical AD dementia based on subtle signs of cognitive impairment, in addition to biomarkers. The combination of biomarker and cognitive markers in this identification process is particularly important because not all people who are positive for AD biomarkers go on to develop dementia,^49^ while cognitive impairment is the source of disability of the disease and the primary threat to public health.

Limitations of this study include neuroimaging at no more than two time points in our data. Nonetheless, the Memento cohort is a cohort study in progress, and one more neuroimaging session is planned for every participant during future follow‐up visits. The Memento cohort is a large and unique collection of individuals who visited a memory clinic, as there was no exclusion based on the cognitive domain of complaints or impairment (eg, isolated non‐memory deficits). This inclusive and non‐selective approach resulted in a real‐life cohort that represents the whole scope of individuals that come to a memory clinic, which is important for generalization purposes. However, due to its nature of being a memory clinic cohort, another limitation is that the Memento cohort only includes individuals with subjective or objective cognitive impairment. This condition prohibited us from comparing the fluency trajectories of these individuals and the relationships of their fluency performance with neurodegeneration to those in cognitively healthy individuals without any subjective or objective cognitive impairment, who may show different trajectories or cognitive‐neural associations. Nonetheless, a strength of the Memento cohort is that it includes sizeable groups of individuals with amnestic and naMCI (in addition to no MCI and incident dementia), which enabled us to compare analyses across these two groups that are known to are more likely to progress to AD versus non‐AD dementia, respectively.^36^

Multiple cross‐sectional studies on semantic and letter fluency that compared different neurodegenerative diseases have shown dissociative patterns in the two verbal fluency tasks, in relation to regional cortical or subcortical damage.^50^, ^51^ In our study, the naMCI group, which is thought to be at higher risk for non‐AD dementia, showed different patterns of associations between verbal fluency tasks at baseline and over time in relation to neurodegeneration compared to the groups at higher risk for AD. Future research should investigate if verbal fluency trajectories also change over time in other neurodegenerative diseases and if so, in which direction and to what extent. This knowledge would also help to clarify to what extent the discrepancy between semantic and letter fluency can be used as a specific marker (in addition to a sensitive marker) for AD compared to other dementias.^52^

In sum, the non‐invasive, low‐cost, classic, and easy‐to‐administer cognitive measure of semantic fluency, and its diverging performance from letter fluency, may aid preclinical detection of AD. This study attests to the value of including semantic and letter fluency as basic tasks in every cognitive assessment in aging research. The discrepancy in semantic and letter fluency performance may be useful in diagnostic prediction of future AD‐related neurodegeneration across multimodal neuroimaging.

## CONFLICTS OF INTEREST

The authors have no relevant conflicts of interest or financial or other nonprofessional benefits to disclose that could bias the authors in the conduct of the reported work.

## AUTHOR CONTRIBUTIONS

Jet M. J. Vonk conceived the idea, performed the analyses, and wrote the manuscript. Vincent Bouteloup helped shape the project idea and execution, and verified the analytical methods. Jean‐François Mangin directs CATI, the platform in charge of Memento's neuroimaging; contributed to imaging analysis; and provided critical feedback. Marie Chupin supervised MRI harmonization, collection, and quality control; contributed to imaging analysis; and provided critical feedback. Marie Odile Habert supervised PET harmonization, collection, and quality control; contributed to imaging analysis; and provided critical feedback. Bruno Dubois, Frédéric Blanc, Audrey Gabelle, Mathieu Ceccaldi, Cédric Annweiller, Pierre Krolak Salmon, Catherine Belin, Thérèse Rivasseau‐Jonveaux, Adrien Jullian, François Sellal, and Eloi Magnin included participants in the cohort and contributed to data collection, and made comments on the manuscript. Geneviève Chêne and Carole Dufouil created and direct the MEMENTO cohort, helped supervise the current project, verified the analytical methods, provided critical feedback, and helped shape the manuscript.

## Supporting information

Supplementary information
