# Supplementary material for: Semantic loss marks early Alzheimer's disease‐related neurodegeneration in older adults without dementia
Source: Alzheimers Dement (Amst). 2020 Aug 5;12(1):e12066. doi: 10.1002/dad2.12066 (PMC7403823; doi:10.1002/dad2.12066)

# Supplemental Materials

Supplementary Table 1. Relationships of verbal fluency performance (baseline and trajectory) with global and regional neurodegeneration over time across groups of cognitive status at last follow-up

|  |  |  | *Baseline fluency* |  | *Change in fluency* |  |
| --- | --- | --- | --- | --- | --- | --- |
|  | Group | Fluency | Unadjusted | Adjusted | Unadjusted | Adjusted |
| Global neurodegeneration | |  |  |  |  |  |
| Total cortical thickness | aMCI vs. no MCI | Semantic | .008 (.005), p = .087 | .008 (.005), p = .081 | .102 (.084), p = .224 | .095 (.084), p = .257 |
|  | naMCI vs. no MCI | Semantic | .007 (.004), p = .110 | .007 (.004), p = .113 | -.194 (.078), p = .013 | -.192 (.078), p = .014 |
|  | dementia vs. no MCI | Semantic | **.014 (.007), p = .040** | **.015 (.007), p = .036** | .233 (.185), p = .210 | .226 (.185), p = .222 |
|  | aMCI vs. naMCI | Semantic | .001 (.004), p = .834 | .001 (.004), p = .794 | **.296 (.080), p < .001** | **.287 (.081), p < .001** |
|  | dementia vs. naMCI | Semantic | .007 (.007), p = .283 | .008 (.007), p = .262 | **.427 (.184), p = .020** | **.418 (.184), p = .023** |
|  | aMCI vs. no MCI | Letter | .002 (.005), p = .641 | .002 (.005), p = .663 | .173 (.115), p = .132 | .165 (.115), p = .151 |
|  | naMCI vs. no MCI | Letter | -.002 (.005), p = .655 | -.002 (.005), p = .654 | **.259 (.110), p = .019** | **.259 (.110), p = .019** |
|  | dementia vs. no MCI | Letter | -.006 (.007), p = .406 | -.006 (.007), p = .407 | .281 (.253), p = .269 | .274 (.254), p = .280 |
|  | aMCI vs. naMCI | Letter | .005 (.005), p = .355 | .005 (.005), p = .370 | -.086 (.115), p = .451 | -.094 (.115), p = .412 |
|  | dementia vs. naMCI | Letter | -.004 (.007), p = .603 | -.004 (.007), p = .605 | .022 (.253), p = .932 | .015 (.253), p = .953 |
| WMH volume | aMCI vs. no MCI | Semantic | < .001 (.003), p = .901 | < .001 (.003), p = .924 | -.022 (.062), p = .722 | -.015 (.062), p = .811 |
|  | naMCI vs. no MCI | Semantic | .003 (.003), p = .388 | .003 (.003), p = .388 | -.105 (.058), p = .069 | -.108 (.058), p = .063 |
|  | dementia vs. no MCI | Semantic | .012 (.005), p = .019 | .012 (.005), p = .021 | .066 (.135), p = .626 | .073 (.135), p = .589 |
|  | aMCI vs. naMCI | Semantic | -.002 (.003), p = .460 | -.002 (.003), p = .442 | .084 (.060), p = .166 | .093 (.060), p = .124 |
|  | dementia vs. naMCI | Semantic | .009 (.005), p = .065 | .009 (.005), p = .069 | .171 (.134), p = .202 | .181 (.134), p = .179 |
|  | aMCI vs. no MCI | Letter | .001 (.004), p = .705 | .002 (.004), p = .663 | .008 (.084), p = .922 | .011 (.084), p = .894 |
|  | naMCI vs. no MCI | Letter | .002 (.004), p = .683 | .002 (.004), p = .679 | -.023 (.080), p = .779 | -.023 (.080), p = .774 |
|  | dementia vs. no MCI | Letter | .004 (.005), p = .412 | .004 (.005), p = .414 | .317 (.182), p = .082 | .319 (.182), p = .080 |
|  | aMCI vs. naMCI | Letter | < .001 (.004), p = .985 | < .001 (.004), p = .972 | .031 (.084), p = .713 | .034 (.084), p = .683 |
|  | dementia vs. naMCI | Letter | .003 (.005), p = .589 | .003 (.005), p = .595 | .339 (.182), p = .062 | .342 (.182), p = .060 |
| ^18^F-FDG-PET SUVR | aMCI vs. no MCI | Semantic | -.014 (.009), p = .127 | -.013 (.009), p = .147 | .137 (.155), p = .379 | .133 (.156), p = .394 |
|  | naMCI vs. no MCI | Semantic | -.004 (.008), p = .642 | -.004 (.008), p = .625 | -.208 (.135), p = .124 | -.207 (.135), p = .125 |
|  | dementia vs. no MCI | Semantic | -.018 (.014), p = .215 | -.017 (.014), p = .225 | -.270 (.365), p = .459 | -.279 (.365), p = .445 |
|  | aMCI vs. naMCI | Semantic | -.010 (.008), p = .218 | -.009 (.008), p = .260 | **.344 (.149), p = .021** | **.340 (.149), p = .023** |
|  | dementia vs. naMCI | Semantic | -.014 (.014), p = .308 | -.014 (.014), p = .328 | -.062 (.362), p = .863 | -.072 (.363), p = .843 |
|  | aMCI vs. no MCI | Letter | -.030 (.010), p = .002 | -.030 (.010), p = .003 | .080 (.225), p = .722 | .061 (.225), p = .786 |
|  | naMCI vs. no MCI | Letter | -.007 (.009), p = .455 | -.007 (.009), p = .459 | -.188 (.197), p = .341 | -.190 (.197), p = .335 |
|  | dementia vs. no MCI | Letter | -.007 (.014), p = .591 | -.008 (.014), p = .576 | -.363 (.475), p = .446 | -.385 (.475), p = .418 |
|  | aMCI vs. naMCI | Letter | -.023 (.009), p = .013 | -.023 (.009), p = .014 | .268 (.223), p = .230 | .251 (.223), p = .261 |
|  | dementia vs. naMCI | Letter | -.001 (.013), p = .965 | -.001 (.013), p = .943 | -.175 (.474), p = .713 | -.195 (.474), p = .681 |
| Regional neurodegeneration | |  |  |  |  |  |
| *Inferior parietal lobule* | aMCI vs. no MCI | Semantic | .004 (.005), p = .432 | .004 (.005), p = .411 | .098 (.098), p = .319 | .090 (.098), p = .360 |
| Cortical thickness | naMCI vs. no MCI | Semantic | .002 (.005), p = .638 | .002 (.005), p = .646 | -.134 (.091), p = .142 | -.132 (.091), p = .149 |
|  | dementia vs. no MCI | Semantic | .012 (.008), p = .151 | .012 (.008), p = .140 | .330 (.217), p = .128 | .323 (.217), p = .137 |
|  | aMCI vs. naMCI | Semantic | .002 (.005), p = .718 | .002 (.005), p = .683 | **.232 (.094), p = .014** | **.222 (.094), p = .019** |
|  | dementia vs. naMCI | Semantic | .009 (.008), p = .243 | .010 (.008), p = .225 | **.464 (.215), p = .031** | **.455 (.215), p = .034** |
|  | aMCI vs. no MCI | Letter | .001 (.006), p = .904 | < .001 (.006), p = .944 | .254 (.134), p = .057 | .237 (.134), p = .076 |
|  | naMCI vs. no MCI | Letter | -.007 (.006), p = .257 | -.007 (.006), p = .256 | **.379 (.128), p = .003** | **.380 (.128), p = .003** |
|  | dementia vs. no MCI | Letter | -.004 (.008), p = .629 | -.004 (.008), p = .631 | .403 (.296), p = .173 | .389 (.295), p = .188 |
|  | aMCI vs. naMCI | Letter | .007 (.006), p = .209 | .007 (.006), p = .229 | -.125 (.133), p = .349 | -.142 (.133), p = .287 |
|  | dementia vs. naMCI | Letter | .003 (.008), p = .738 | .003 (.008), p = .735 | .024 (.295), p = .935 | .010 (.295), p = .974 |
| *Inferior parietal lobule* | aMCI vs. no MCI | Semantic | < .001 (.009), p = .986 | < .001 (.009), p = .969 | .116 (.163), p = .476 | .112 (.163), p = .495 |
| ^18^F-FDG-PET SUVR^a^ | naMCI vs. no MCI | Semantic | -.002 (.008), p = .836 | -.002 (.008), p = .821 | -.063 (.141), p = .659 | -.062 (.142), p = .661 |
|  | dementia vs. no MCI | Semantic | -.006 (.015), p = .674 | -.006 (.015), p = .689 | .489 (.383), p = .201 | .479 (.383), p = .212 |
|  | aMCI vs. naMCI | Semantic | .002 (.008), p = .858 | .002 (.009), p = .796 | .179 (.156), p = .252 | .174 (.156), p = .267 |
|  | dementia vs. naMCI | Semantic | -.005 (.014), p = .751 | -.004 (.014), p = .776 | .552 (.379), p = .146 | .541 (.380), p = .155 |
|  | aMCI vs. no MCI | Letter | -.005 (.010), p = .644 | -.006 (.010), p = .585 | .014 (.235), p = .951 | -.004 (.236), p = .988 |
|  | naMCI vs. no MCI | Letter | -.001 (.009), p = .937 | -.001 (.009), p = .922 | .035 (.207), p = .867 | .033 (.207), p = .875 |
|  | dementia vs. no MCI | Letter | .006 (.014), p = .683 | .007 (.014), p = .629 | .561 (.497), p = .260 | .539 (.498), p = .280 |
|  | aMCI vs. naMCI | Letter | -.004 (.010), p = .681 | -.005 (.010), p = .630 | -.02 (.233), p = .932 | -.036 (.234), p = .877 |
|  | dementia vs. naMCI | Letter | .007 (.014), p = .634 | .008 (.014), p = .571 | .527 (.495), p = .288 | .506 (.496), p = .308 |
| *Pars opercularis* | aMCI vs. no MCI | Semantic | .010 (.006), p = .079 | .010 (.006), p = .083 | .075 (.101), p = .458 | .067 (.101), p = .507 |
| Cortical thickness | naMCI vs. no MCI | Semantic | .009 (.005), p = .091 | .009 (.005), p = .090 | -.116 (.095), p = .222 | -.113 (.095), p = .232 |
|  | dementia vs. no MCI | Semantic | .016 (.008), p = .056 | .016 (.008), p = .060 | .175 (.224), p = .434 | .168 (.224), p = .454 |
|  | aMCI vs. naMCI | Semantic | .001 (.005), p = .863 | .001 (.005), p = .891 | **.191 (.097), p = .050** | .181 (.098), p = .064 |
|  | dementia vs. naMCI | Semantic | .007 (.008), p = .388 | .007 (.008), p = .408 | .291 (.222), p = .190 | .281 (.222), p = .205 |
|  | aMCI vs. no MCI | Letter | .006 (.006), p = .317 | .006 (.006), p = .336 | .053 (.138), p = .700 | .063 (.139), p = .649 |
|  | naMCI vs. no MCI | Letter | -.002 (.006), p = .746 | -.002 (.006), p = .745 | .216 (.133), p = .103 | .216 (.133), p = .104 |
|  | dementia vs. no MCI | Letter | -.006 (.009), p = .465 | -.006 (.009), p = .467 | .179 (.306), p = .559 | .187 (.306), p = .542 |
|  | aMCI vs. naMCI | Letter | .008 (.006), p = .176 | .008 (.006), p = .188 | -.163 (.138), p = .238 | -.153 (.138), p = .269 |
|  | dementia vs. naMCI | Letter | -.004 (.008), p = .612 | -.004 (.008), p = .615 | -.038 (.306), p = .902 | -.029 (.306), p = .923 |
| *Pars opercularis* | aMCI vs. no MCI | Semantic | .006 (.008), p = .477 | .006 (.008), p = .487 | -.065 (.146), p = .656 | -.059 (.146), p = .686 |
| ^18^F-FDG-PET SUVR | naMCI vs. no MCI | Semantic | -.001 (.007), p = .931 | -.001 (.007), p = .935 | -.095 (.126), p = .452 | -.095 (.126), p = .450 |
|  | dementia vs. no MCI | Semantic | .007 (.013), p = .602 | .007 (.013), p = .606 | .318 (.342), p = .353 | .332 (.343), p = .333 |
|  | aMCI vs. naMCI | Semantic | .007 (.008), p = .389 | .006 (.008), p = .402 | .030 (.139), p = .828 | .036 (.140), p = .794 |
|  | dementia vs. naMCI | Semantic | .008 (.013), p = .556 | .007 (.013), p = .561 | .413 (.339), p = .224 | .428 (.340), p = .209 |
|  | aMCI vs. no MCI | Letter | .003 (.009), p = .712 | .003 (.009), p = .744 | -.062 (.211), p = .769 | -.089 (.211), p = .675 |
|  | naMCI vs. no MCI | Letter | .001 (.009), p = .880 | .001 (.009), p = .890 | .003 (.184), p = .985 | < .001 (.184), p = > .999 |
|  | dementia vs. no MCI | Letter | .006 (.013), p = .628 | .007 (.013), p = .601 | .361 (.445), p = .418 | .327 (.445), p = .462 |
|  | aMCI vs. naMCI | Letter | .002 (.009), p = .807 | .002 (.009), p = .834 | -.065 (.208), p = .754 | -.089 (.208), p = .671 |
|  | dementia vs. naMCI | Letter | .005 (.012), p = .691 | .006 (.012), p = .655 | .357 (.443), p = .420 | .327 (.443), p = .460 |
| *Hippocampal volume* | aMCI vs. no MCI | Semantic | .005 (.003), p = .101 | .005 (.003), p = .096 | **.099 (.050), p = .050** | .096 (.050), p = .058 |
|  | naMCI vs. no MCI | Semantic | .002 (.003), p = .349 | .002 (.003), p = .354 | -.028 (.047), p = .551 | -.027 (.047), p = .563 |
|  | dementia vs. no MCI | Semantic | -.004 (.004), p = .296 | -.004 (.004), p = .312 | .002 (.111), p = .988 | -.001 (.111), p = .992 |
|  | aMCI vs. naMCI | Semantic | .002 (.003), p = .427 | .002 (.003), p = .406 | **.127 (.048), p = .009** | **.123 (.049), p = .012** |
|  | dementia vs. naMCI | Semantic | -.007 (.004), p = .094 | -.007 (.004), p = .102 | .030 (.110), p = .787 | .026 (.110), p = .813 |
|  | aMCI vs. no MCI | Letter | -.001 (.003), p = .802 | -.001 (.003), p = .809 | .009 (.069), p = .892 | .004 (.069), p = .948 |
|  | naMCI vs. no MCI | Letter | .001 (.003), p = .839 | .001 (.003), p = .839 | -.022 (.066), p = .738 | -.022 (.066), p = .738 |
|  | dementia vs. no MCI | Letter | -.002 (.004), p = .621 | -.002 (.004), p = .621 | -.015 (.152), p = .922 | -.020 (.152), p = .897 |
|  | aMCI vs. naMCI | Letter | -.001 (.003), p = .645 | -.001 (.003), p = .651 | .031 (.069), p = .648 | .026 (.069), p = .700 |
|  | dementia vs. naMCI | Letter | -.003 (.004), p = .516 | -.003 (.004), p = .515 | .007 (.152), p = .962 | .002 (.152), p = .988 |
| *Entorhinal cortex* | aMCI vs. no MCI | Semantic | .002 (.004), p = .635 | .002 (.004), p = .625 | .130 (.067), p = .053 | **.132 (.067), p = .049** |
| Cortical thickness | naMCI vs. no MCI | Semantic | -.004 (.004), p = .243 | -.004 (.004), p = .241 | .009 (.063), p = .883 | .009 (.063), p = .891 |
|  | dementia vs. no MCI | Semantic | .003 (.006), p = .585 | .003 (.006), p = .574 | .080 (.148), p = .591 | .082 (.148), p = .580 |
|  | aMCI vs. naMCI | Semantic | .006 (.003), p = .095 | .006 (.004), p = .091 | .121 (.064), p = .061 | .123 (.065), p = .056 |
|  | dementia vs. naMCI | Semantic | .007 (.005), p = .189 | .007 (.005), p = .182 | .071 (.147), p = .631 | .073 (.147), p = .618 |
|  | aMCI vs. no MCI | Letter | .001 (.004), p = .756 | .001 (.004), p = .805 | .047 (.092), p = .605 | .040 (.092), p = .660 |
|  | naMCI vs. no MCI | Letter | .003 (.004), p = .466 | .003 (.004), p = .467 | .019 (.088), p = .830 | .019 (.088), p = .828 |
|  | dementia vs. no MCI | Letter | -.008 (.006), p = .177 | -.008 (.006), p = .179 | -.078 (.203), p = .702 | -.085 (.203), p = .675 |
|  | aMCI vs. naMCI | Letter | -.002 (.004), p = .683 | -.002 (.004), p = .637 | .029 (.091), p = .755 | .021 (.092), p = .816 |
|  | dementia vs. naMCI | Letter | -.011 (.006), p = .057 | -.011 (.006), p = .058 | -.097 (.202), p = .633 | -.104 (.202), p = .607 |
| *Parahippocampal gyrus* | aMCI vs. no MCI | Semantic | .003 (.003), p = .251 | .003 (.003), p = .248 | .086 (.053), p = .103 | .085 (.053), p = .111 |
| Cortical thickness | naMCI vs. no MCI | Semantic | < .001 (.003), p = > .999 | < .001 (.003), p = .998 | -.004 (.050), p = .936 | -.004 (.050), p = .943 |
|  | dementia vs. no MCI | Semantic | .002 (.004), p = .699 | .002 (.004), p = .693 | .020 (.117), p = .862 | .019 (.117), p = .874 |
|  | aMCI vs. naMCI | Semantic | .003 (.003), p = .225 | .003 (.003), p = .221 | .090 (.051), p = .075 | .088 (.051), p = .084 |
|  | dementia vs. naMCI | Semantic | .002 (.004), p = .692 | .002 (.004), p = .684 | .024 (.116), p = .834 | .022 (.116), p = .850 |
|  | aMCI vs. no MCI | Letter | .001 (.003), p = .837 | .001 (.003), p = .793 | .114 (.073), p = .116 | .104 (.073), p = .152 |
|  | naMCI vs. no MCI | Letter | -.003 (.003), p = .416 | -.003 (.003), p = .416 | .115 (.069), p = .098 | .115 (.069), p = .097 |
|  | dementia vs. no MCI | Letter | -.005 (.005), p = .243 | -.005 (.005), p = .240 | .086 (.160), p = .592 | .077 (.160), p = .631 |
|  | aMCI vs. naMCI | Letter | .003 (.003), p = .306 | .003 (.003), p = .280 | -.001 (.072), p = .992 | -.011 (.072), p = .877 |
|  | dementia vs. naMCI | Letter | -.003 (.004), p = .551 | -.003 (.004), p = .546 | -.029 (.160), p = .855 | -.038 (.160), p = .810 |
| *Inferior temporal area^a^* | aMCI vs. no MCI | Semantic | .004 (.009), p = .636 | .005 (.009), p = .602 | -.028 (.161), p = .863 | -.028 (.162), p = .862 |
| ^18^F-FDG-PET SUVR | naMCI vs. no MCI | Semantic | .005 (.008), p = .542 | .005 (.008), p = .553 | -.102 (.140), p = .466 | -.102 (.140), p = .466 |
|  | dementia vs. no MCI | Semantic | -.026 (.015), p = .083 | -.025 (.015), p = .086 | .394 (.378), p = .298 | .393 (.379), p = .300 |
|  | aMCI vs. naMCI | Semantic | -.001 (.009), p = .951 | < .001 (.009), p = .994 | .074 (.154), p = .630 | .074 (.155), p = .632 |
|  | dementia vs. naMCI | Semantic | -.031 (.014), p = .033 | -.030 (.014), p = .035 | .496 (.375), p = .186 | .495 (.376), p = .188 |
|  | aMCI vs. no MCI | Letter | -.008 (.010), p = .438 | -.008 (.010), p = .430 | .218 (.233), p = .350 | .207 (.234), p = .376 |
|  | naMCI vs. no MCI | Letter | .006 (.009), p = .551 | .006 (.009), p = .554 | -.039 (.204), p = .849 | -.040 (.204), p = .843 |
|  | dementia vs. no MCI | Letter | -.030 (.014), p = .038 | -.030 (.014), p = .039 | .649 (.491), p = .187 | .635 (.492), p = .197 |
|  | aMCI vs. naMCI | Letter | -.014 (.010), p = .161 | -.014 (.01), p = .158 | .257 (.231), p = .268 | .247 (.232), p = .287 |
|  | dementia vs. naMCI | Letter | -.035 (.014), p = .011 | -.035 (.014), p = .011 | .688 (.490), p = .160 | .676 (.490), p = .169 |
| *Isthmus cingulate* | aMCI vs. no MCI | Semantic | .002 (.004), p = .549 | .002 (.004), p = .519 | **.134 (.068), p = .048** | .132 (.068), p = .052 |
| Cortical thickness | naMCI vs. no MCI | Semantic | **.008 (.004), p = .019** | **.008 (.004), p = .020** | -.040 (.063), p = .525 | -.040 (.063), p = .531 |
|  | dementia vs. no MCI | Semantic | -.001 (.006), p = .928 | < .001 (.006), p = .969 | .071 (.150), p = .637 | .069 (.150), p = .647 |
|  | aMCI vs. naMCI | Semantic | -.006 (.004), p = .088 | -.006 (.004), p = .101 | **.175 (.065), p = .007** | **.172 (.065), p = .009** |
|  | dementia vs. naMCI | Semantic | -.009 (.005), p = .108 | -.008 (.006), p = .123 | .111 (.148), p = .455 | .108 (.149), p = .466 |
|  | aMCI vs. no MCI | Letter | .001 (.004), p = .899 | < .001 (.004), p = .946 | .021 (.093), p = .818 | .017 (.093), p = .856 |
|  | naMCI vs. no MCI | Letter | -.002 (.004), p = .574 | -.002 (.004), p = .572 | .048 (.089), p = .591 | .048 (.089), p = .590 |
|  | dementia vs. no MCI | Letter | < .001 (.006), p = .992 | < .001 (.006), p = .987 | .231 (.205), p = .259 | .227 (.205), p = .268 |
|  | aMCI vs. naMCI | Letter | .003 (.004), p = .489 | .003 (.004), p = .527 | -.027 (.093), p = .774 | -.031 (.093), p = .738 |
|  | dementia vs. naMCI | Letter | .002 (.006), p = .674 | .002 (.006), p = .668 | .184 (.205), p = .370 | .180 (.205), p = .381 |
| *Precuneus–posterior cingulate area^a^* | aMCI vs. no MCI | Semantic | < .001 (.008), p = .980 | .001 (.008), p = .915 | -.031 (.141), p = .824 | -.036 (.141), p = .798 |
|  | naMCI vs. no MCI | Semantic | .004 (.007), p = .589 | .004 (.007), p = .606 | -.127 (.122), p = .298 | -.127 (.122), p = .300 |
| ^18^F-FDG-PET SUVR | dementia vs. no MCI | Semantic | -.014 (.013), p = .263 | -.014 (.013), p = .276 | .137 (.331), p = .678 | .126 (.331), p = .704 |
|  | aMCI vs. naMCI | Semantic | -.004 (.007), p = .626 | -.003 (.007), p = .709 | .096 (.135), p = .477 | .091 (.135), p = .502 |
|  | dementia vs. naMCI | Semantic | -.018 (.012), p = .143 | -.018 (.012), p = .155 | .264 (.328), p = .420 | .253 (.328), p = .442 |
|  | aMCI vs. no MCI | Letter | -.005 (.009), p = .590 | -.006 (.009), p = .522 | .090 (.203), p = .656 | .075 (.204), p = .714 |
|  | naMCI vs. no MCI | Letter | .001 (.008), p = .867 | .001 (.008), p = .884 | .059 (.178), p = .743 | .057 (.178), p = .750 |
|  | dementia vs. no MCI | Letter | -.017 (.012), p = .178 | -.016 (.012), p = .206 | .666 (.430), p = .122 | .646 (.431), p = .134 |
|  | aMCI vs. naMCI | Letter | -.006 (.008), p = .464 | -.007 (.008), p = .413 | .032 (.201), p = .874 | .018 (.202), p = .929 |
|  | dementia vs. naMCI | Letter | -.018 (.012), p = .132 | -.017 (.012), p = .160 | .608 (.428), p = .157 | .589 (.429), p = .170 |

*Note*. Cells represent beta estimate (standard error), p-value—values in bold represent that a lower baseline fluency performance or faster rate of fluency decline related to more neurodegeneration; ^a^Disease-specific 18F-FDG-PET SUVR ROIs inferred from the ADNI database [31]; MCI = mild cognitive impairment, aMCI = amnestic MCI, naMCI = non-amnestic MCI; ROIs are in the left hemisphere; ^18^F-FDG-PET = ^18^F-fluorodeoxyglucose brain positron emission tomography; SUVR = Standardized Uptake Value Ratio; models are adjusted for age, sex, and education

Supplementary Figure 1. Relationships of rate of change in semantic and letter fluency with neurodegeneration


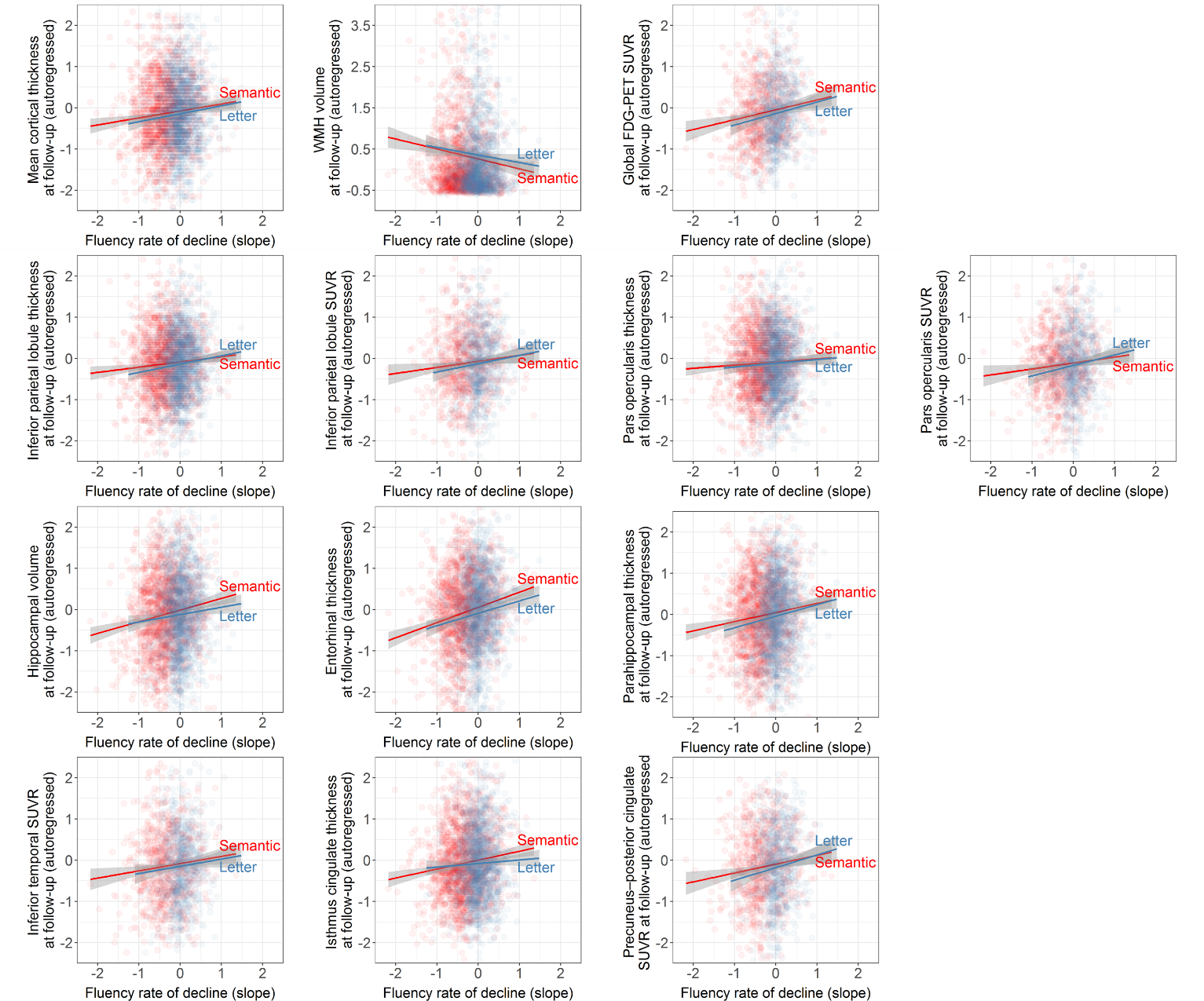

Supplement: Supplementary file 1 — Supplementary information [file DAD2-12-e12066-s001.docx]
